# Analyzing Healthcare Facility Resilience: Scientometric Review and Knowledge Map

**DOI:** 10.3389/fpubh.2021.764069

**Published:** 2021-11-08

**Authors:** Lingzhi Li, Shuni Liao, Jingfeng Yuan, Endong Wang, Jianjun She

**Affiliations:** ^1^Research Center of Smart City, Nanjing Tech University, Nanjing, China; ^2^Department of Construction and Real Estate, School of Civil Engineering, Southeast University, Nanjing, China; ^3^Department of Sustainable Resources Management, State University of New York, Syracuse, NY, United States

**Keywords:** healthcare facility, hospitals, resilience, bibliometrics, delivery of health care, knowledge map, epidemics, disasters

## Abstract

In contemporary “high-risk” society, unexpected disasters (epidemics and extreme weather) and chronic pressures (aging problems) put tremendous pressure on healthcare facilities. Enhancing the healthcare facilities' resilience ability to resist, absorb, and respond to disaster disruptions is urgent. This study presents a scientometric review for healthcare facility resilience research. A total of 374 relevant articles published between 2000 and 2020, collected from Web of Science (WoS) core collection database, Scopus database and MEDLINE database were reviewed and analyzed. The results indicated that research on resilience in healthcare facilities went through three development periods, and the research involved countries or institutions that are relatively scattered. The studies have been focused on the subject categories of engineering, public, environmental, and occupational health. The keywords of “resilience,” “hospital,” “disaster,” “healthcare,” and “healthcare facility” had the most frequency. Furthermore, based on the literature co-citation networks and content analysis, the detected seven co-citation clusters were grouped into four knowledge domains: climate change impact, strengthening resilience in response to war and epidemic, resilience assessment of healthcare facility, and the applications of information system. Moreover, the timeline view of literature reflected the evolution of each domain. Finally, a knowledge map for resilience of healthcare facilities was put forward, in which critical research contents, current knowledge gaps, and future research work were discussed. This contribution will promote researchers and practitioners to detect the hot topics, fill the knowledge gaps, and extend the body of research on resilience of healthcare facilities.

## Introduction

In a modern “high-risk” society, the aging population is increasing at an astonishing rate, alongside many disasters that frequently occur, such as earthquakes, floods, hurricanes, and epidemics (1, 2). During disasters, healthcare facilities are critical emergency response resources because they are central to providing timely and good quality healthcare services for the injuries (3, 4). With a broader view, the healthcare facilities are regarded as an elaborate network of buildings, services and relevant public and private sectors for providing and delivering healthcare service for the general public (5), including the national/regional healthcare systems and the single healthcare facilities (e.g., hospitals, clinics and community care centers) (3). A single healthcare facility is composed of a set of interdependent components, such as medical staff, medical resources, medical equipment, physical building structures and equipment systems [e.g., “heating, ventilation, and air conditioning (HVAC) system,” elevators, and power systems] (6). The increasing healthcare demand and disaster events threaten healthcare facilities' functionality (7–11). In such a situation, healthcare facilities are expected to maintain or even increase their capacity to provide continuous healthcare service even if they are directly affected by disasters. Recently, the concept of “healthcare facility resilience” (HFR) has been highlighted in the disaster management lexicon. It's the ability to prepare for, manage (absorb and adapt) and learn from shocks (12). This concept provides a new thinking paradigm for facilitating the healthcare facilities' sustainable operation in the face of disaster disruptions.

Over the past decade, several articles have reviewed HFR-related research. Several scholars have discussed some of the hazards or disturbances that healthcare facilities may face. For example, from a management perspective, Hugelius et al. pointed out that the healthcare facility may suffer, such as the lack of information and resources to deal with mass casualties (13). In 2017, Curtis et al. focused on the impact of extreme weather, such as heat waves, cold waves, and floods, on healthcare facilities and health services in the UK (14). Some other scholars reviewed existing literature and discussed models that could be used to measure HFR. These models may be comprehensive scoring framework models for evaluating some key capabilities of hospitals (15). Conceptual models may also be used to display resilience, such as state-space, stress-strain curve, temporal dynamic, stretched systems, and variety-space (16). Some scholars have been concerned about the response of healthcare facilities to disturbances. For instance, Kost et al. discussed combining “geospatial point-of-care testing” to address public health challenges (17). The needs of the patient are often an essential consideration when responding (16). Recently, more scholars have focused on HFR, especially in the case of the COVID-19 crisis. Haldane et al. reviewed national primary care guidelines for responding to COVID-19 and discussed how they support the operation of healthcare facilities (18).

Although a few articles have reviewed HFR-related studies, most are limited to specific research content. For example, some of these articles focus on evaluating HFR (19), and some are limited to the influence of specific factors on HFR (20). Most of the literature relies on subjective judgment rather than quantitative methods to identify the research topic (14, 19, 21, 22). Therefore, conducting a comprehensive review of HFR-related literature using a quantitative and objective method is necessary. The scientometric analysis is a quantitative method, referred to as knowledge domain visualization and mapping, which provides a holistic view of a particular domain through analyzing published articles (23). Combined with thematic analysis, this study aims to use this technique to review and visualize HFR research systematically from 2000 to 2020. The analysis of countries, institutions, published years, keywords, and subject categories can help understand the overall research status. Combined analysis of citing articles (the selected articles) and cited articles (the references of citing articles) can help identify research hotspots and research frontiers. Citation analysis visualization technology displays the research status and evolution of the knowledge domain on the network map. Finally, a knowledge map of HFR that reveals the critical research parts, knowledge gaps, future directions was proposed.

## Methodology

**Figure 1:**
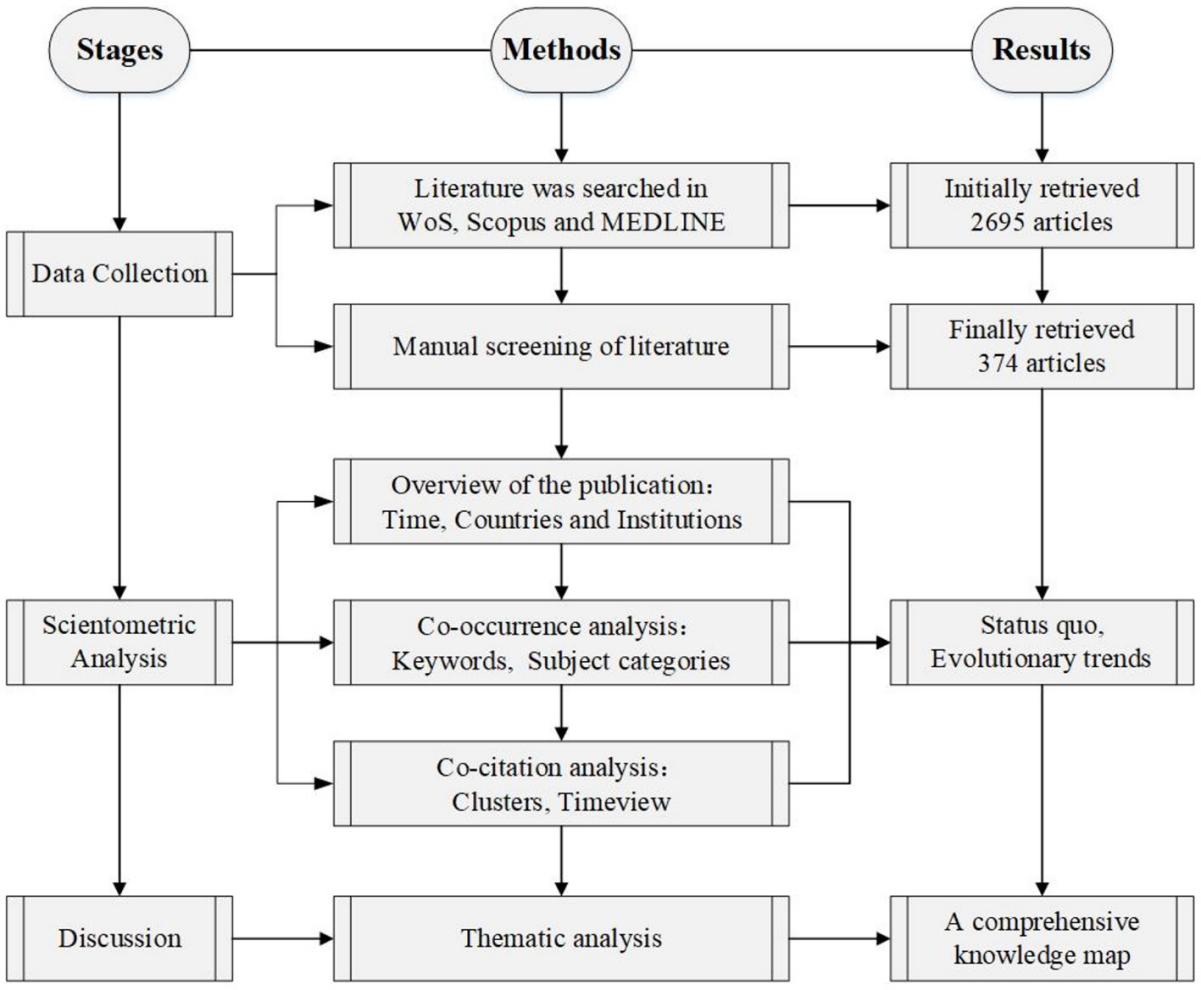
Research framework.

The research framework of this study is shown in Figure 1, which is divided into three steps.

The first step aims to collect enough relevant articles related to HFR research. As one of the most authoritative publication databases, Web of Science (WoS) core collection database, Scopus database and MEDLINE database were selected as the sources for scientometric analysis (24, 25). The searching formula were used to determine more appropriate articles, according to article types and criteria. Following that, the abstracts of the retrieved articles were thoroughly read, and the literatures were screened according to certain criteria.

As the size and scope of HFR studies have expanded, conducting manual scientometric analyses is almost impossible. As a convenient scientometric and visual analytic tool, CiteSpace software able to review the classic research theme and discover potential trends (26, 27). Thus, in the second step of this study, CiteSpace software 5.8.R1 is used to conduct scientometric analysis, including literature distribution analysis, co-occurrence analysis, and co-citation analysis. Critically, the research hotspot and research trend are discussed in each identified knowledge domain.

The final step is to develop the knowledge map of HFR studies, in which the critical research content and knowledge gaps will be discussed.

## Data Collection

Detailed literature retrieval rules and exclusion strategies are as follows.

### Literature Retrieval

The databases searched in WoS include WoS core collection databases (including Science Citation Index Expanded, Social Sciences Citation Index, Arts and Humanities Citation Index and Emerging Sources Citation Index) and MEDLINE database. After pre-analysis and comparison, the determined search schema for searching the WoS core collection database and MEDLINE database was as follows: TS = (resilien^*^) AND TS = (hospital^*^ OR medical OR health^*^ OR care) AND TS = (facility OR facilities OR asset^*^ OR “built environment” OR “building portfolio” OR lifeline OR equipment OR device^*^). “TS” means the topic of an article, and “^*^” refers to a fuzzy search. In addition, the determined search schema for searching the Scopus database was as follows: TITLE-ABS-KEY (resilien^*^) AND TITLE-ABS-KEY (hospital^*^ OR medical OR health^*^ OR care) AND TITLE-ABS-KEY (facility OR facilities OR asset^*^ OR lifeline OR “built environment” OR “building portfolio” OR equipment OR device^*^). Literature published before 2020 (including 2020) was searched from these three databases. The language is limited to English, and the type is confined to article. After data deduplication, 2,695 articles were obtained.

### Exclusion Criteria

The retrieved results need to be reviewed to ensure that the selected articles meet the requirements for further analysis (28, 29). By reading the abstract, unrelated literature was excluded from detailed review and analysis according to the following criteria:

(1) Journals that have not been peer-reviewed will be excluded.
(2) Articles lacking references, authors, and the full text will be excluded.
(3) Repeated articles published in different journals with the same authorship will be excluded (only the oldest ones are retained).
(4) The articles focused on resilience but not the healthcare facility. These articles target psychological resilience (30), supply chain resilience (31), and the resilience of ecosystem (20).
(5) The terms in the search schema are used in different settings or had other irrelevant meanings. For example, “resilience” in Yin et al. (32) did not refer to the capability to respond disasters, maintain its most essential functions, and “bounce back” to the pre-event state (termed recovery) or to a new state of function (termed adaptation) (21) but described how the object moves or deforms under the action of external force.
(6) These articles focus on the research and development of medical devices or technologies to support healthcare services, but HFR is not directly related (33).

By further screening, 374 articles were eventually selected for scientometric analysis, among which there are 251 articles from the WoS core Collection database, 72 from Scopus database, and 51 from MEDLINE database. The period ranges from 2001 to 2020.

## Scientometric Analysis and Results

### Overview of the Publication Year

**Figure 2:**
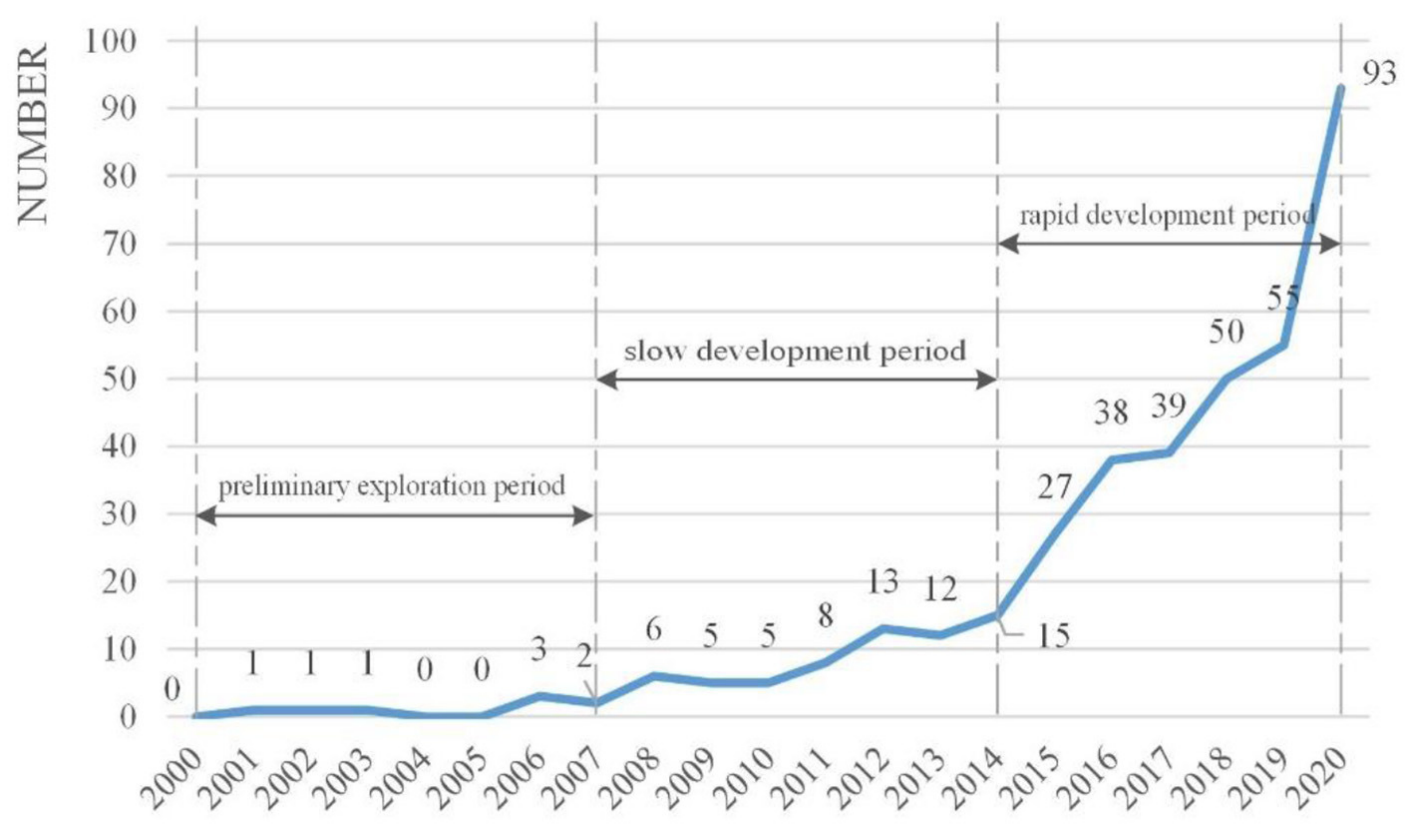
Number of articles on HFR.

Figure 2 shows the distribution of HFR-related articles over time from 2000 to 2020, illustrating that HFR research was thriving. In 2000–2007 the number of published articles (8 articles in total) was relatively small. Preliminary explorations were conducted during this period. In 2008–2014, the number of articles fluctuated between 5 and 15 (64 articles). This period belongs to the period of slow growth. Between 2015 and 2020 is the period of rapid growth, in which the number of published articles grew rapidly from 27 to 93. The number of articles during this period is 302, accounting for 80.75% of the total number of articles (374).

### Overview of the Publication Countries and Publication Institutions

**Figure 3:**
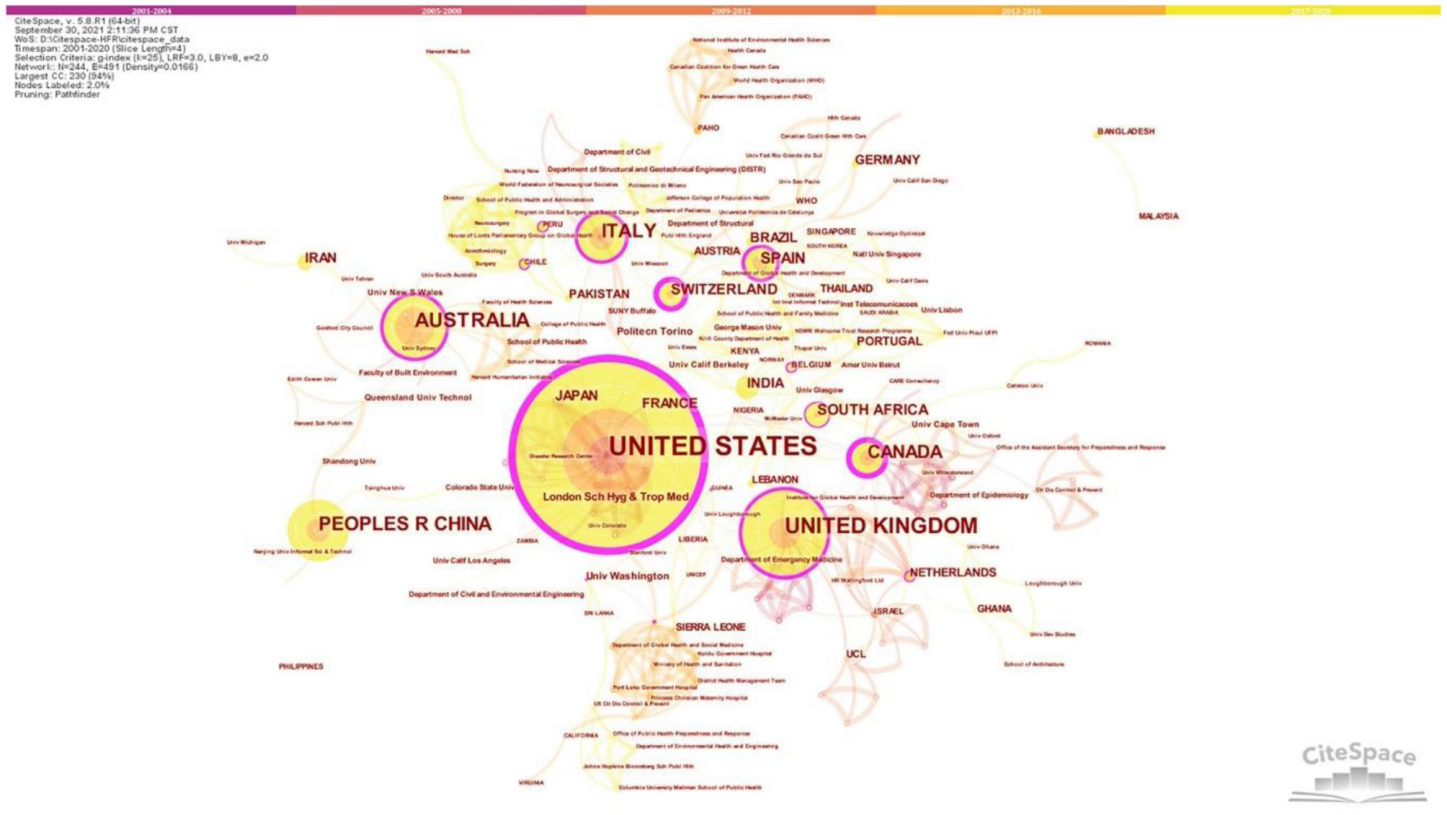
Countries and institutions co-occurrence network.

Figure 3 shows the countries and institutions' co-occurrence network diagram of HFR research. The network contains 244 nodes and 491 links. Among which, 138 nodes represent countries, and 106 nodes display institutions. The larger the node, the more articles are published in the country or institution (34). The top five are the United States (174, accounting for 46.52% of the total), the United Kingdom (67, constituting 19.91% of the total), Australia (41, occupying 10.96% of the total), Italy (36, taking up 9.63% of the total), and China (35, amounting to 9.36% of the total). It is apparent to see that the United States is far ahead in this field. The complex links between nodes indicate a common phenomenon of transnational cooperation in this area. The thicker the link, the more collaborations and the closer the connection between the countries. The purple circle shows that the United States, the United Kingdom, Canada, and Spain have played a crucial role in international cooperation. In general, the distribution of institutions is consistent with the distribution of countries (regions) (35). The United States has the most active research institutions on HFR, University of Washington (6). It is followed by London School of Hygiene and Tropical Medicine (5) and Politeco di Torino (5), which come from the United Kingdom and Italy with high publication outputs, respectively. Overall, institutions conducting and publishing HFR research are relatively scattered.

### Subject Categories Co-occurrence Network

**Figure 4:**
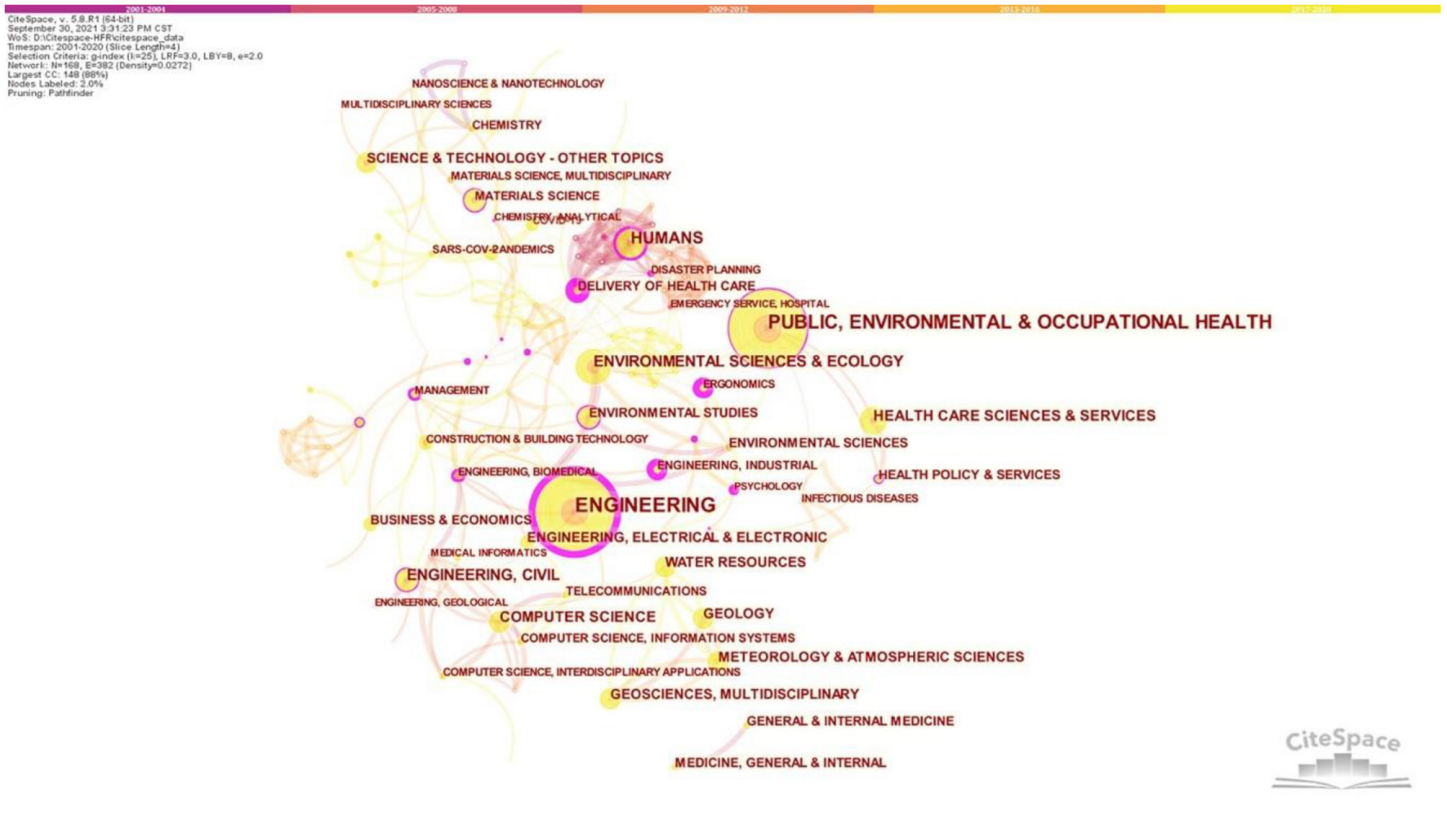
Subject categories co-occurrence network.

The subject category co-occurrence network is shown in Figure 4. The network consists of 168 nodes and 382 links, suggesting that HFR-related research covers 168 subject categories, illustrating that the research is interdisciplinary. The more articles were published in a particular subject area, the larger the node (36). The top five subject categories are engineering (65); public, environmental, and occupational health (63); humans (33); environmental sciences and ecology (28); engineering and civil science (23). In Figure 4, the link between nodes indicates that two topics appear at the same time in the same article. The thicker the link, the greater the frequency. In Figure 4, nodes with high centrality are marked by purple rings (35). Some nodes with high centrality in co-occurring subject categories network are delivery of health care (0.83), ergonomics (0.78), industrial (0.77), and engineering (0.66), which demonstrate that these subject categories are the major turning nodes linking the HFR research in different phases (35).

### Keywords Co-occurrence Network

**Figure 5:**
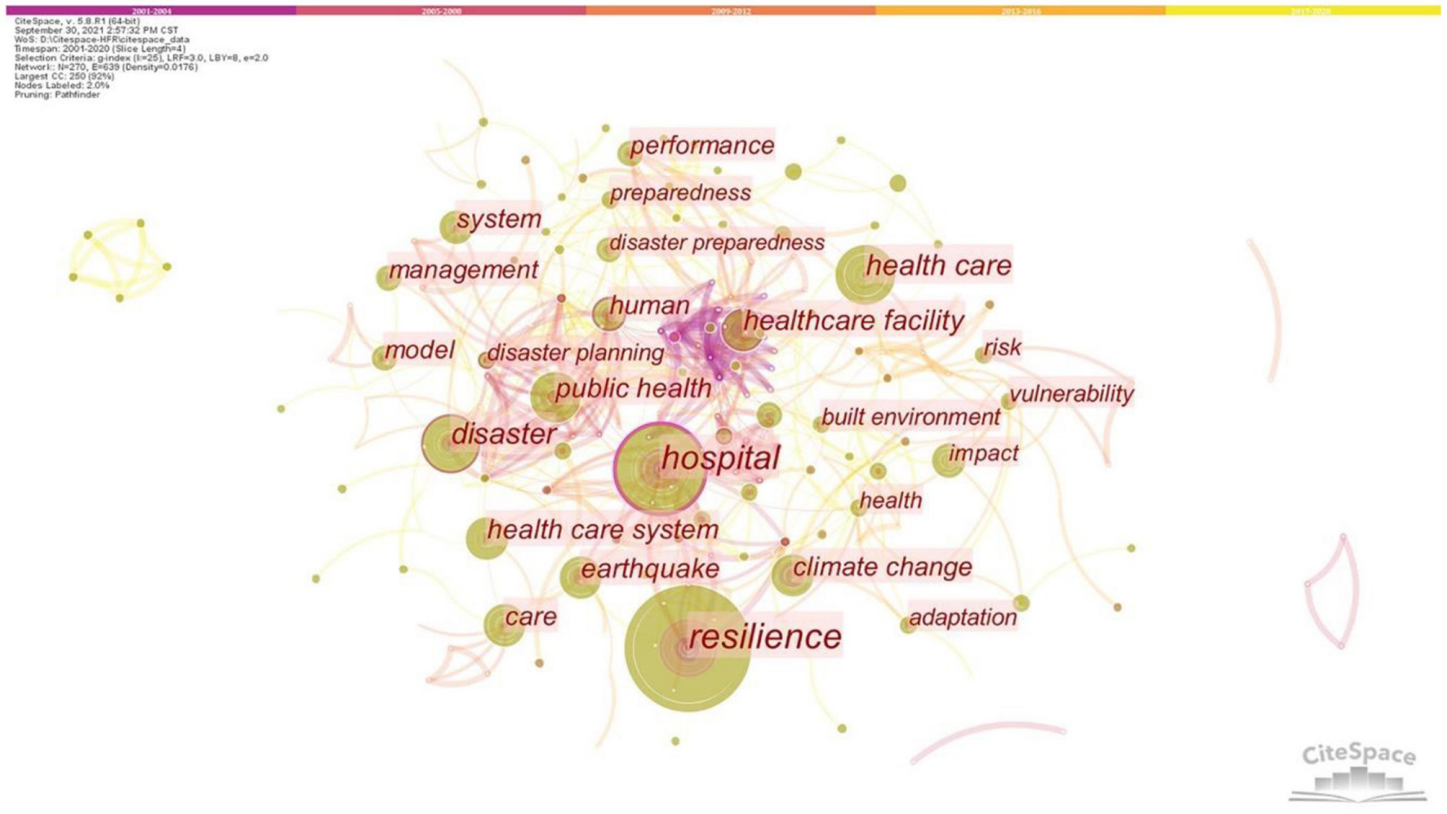
The co-occurrence network of keywords.

According to the articles selected from the two databases, the co-occurrence network of keywords was generated, as shown in Figure 5. This network contains 270 nodes and 639 links, which shows 270 keywords. Node size indicates keyword frequency. The top five high-frequency keywords are “resilience” (frequency = 86), “hospital” (frequency = 63), “disaster” (frequency = 41), “healthcare” (frequency = 37), and “healthcare facility” (frequency = 31). The links between nodes are complicated. Multiple keywords frequently appear together in the same literature and are closely interrelated.

### Literature Co-citation Network and Timeline View

**Figure 6:**
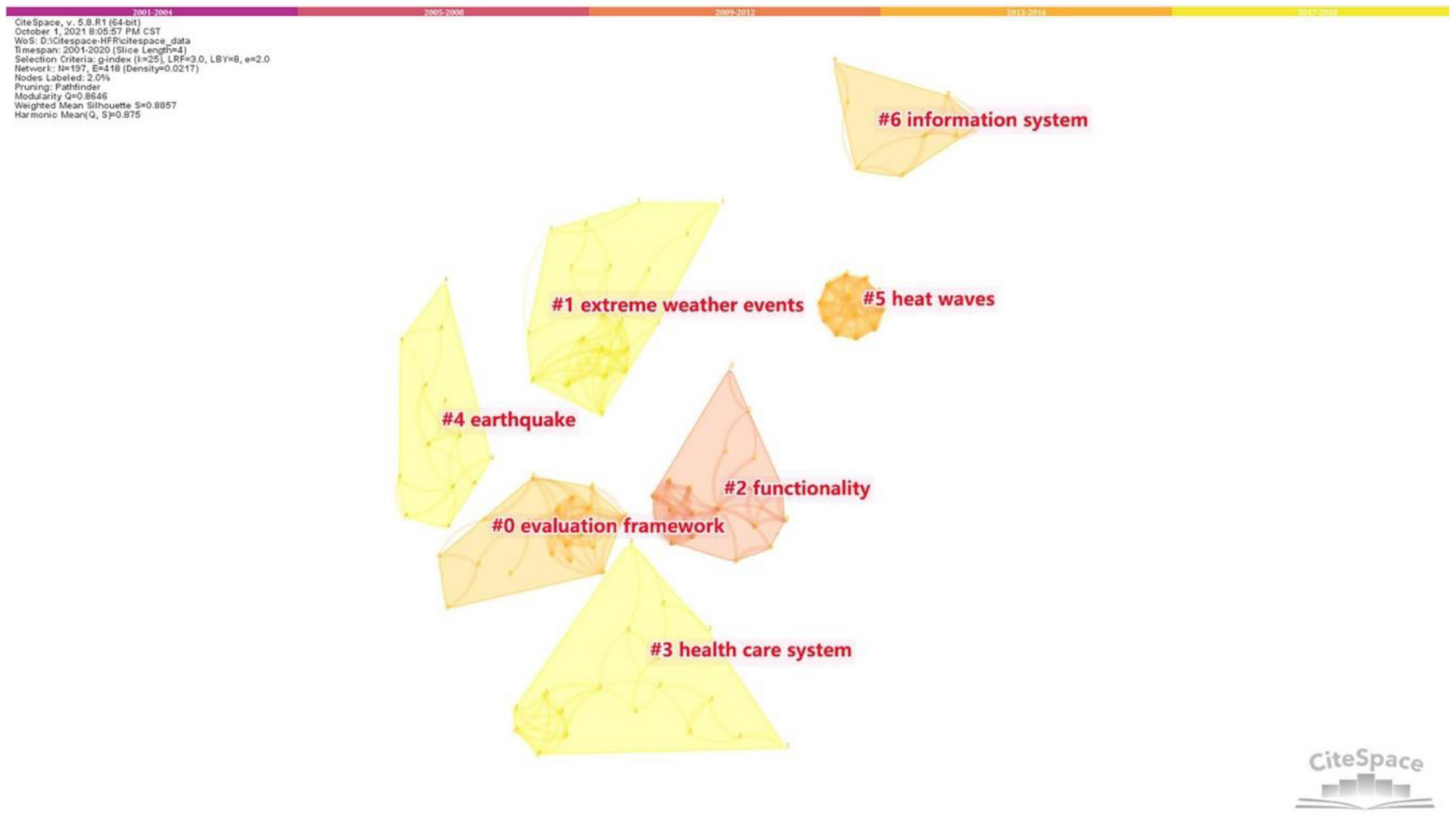
Literature co-citation network.

This study used the LLR algorithm to generate the literature co-cited network (Figure 6). This network consists of 197 nodes and 418 links. The modularity is 0.8646, indicating that these co-citation clusters can define the research areas of HFR (37). The mean silhouette is 0.8857 (>0.5), so the clustering result is reasonable (26). The silhouette of the major clusters discussed in the study is all over 0.9, reflecting the high homogeneity of the network (38). After excluding the small clusters with a small number of articles (the number <10), 7 clusters (#0 – #6) were finally identified in the literature co-cited network. The label of each cluster was determined by the citing literature in the cluster (26).

**Figure 7:**
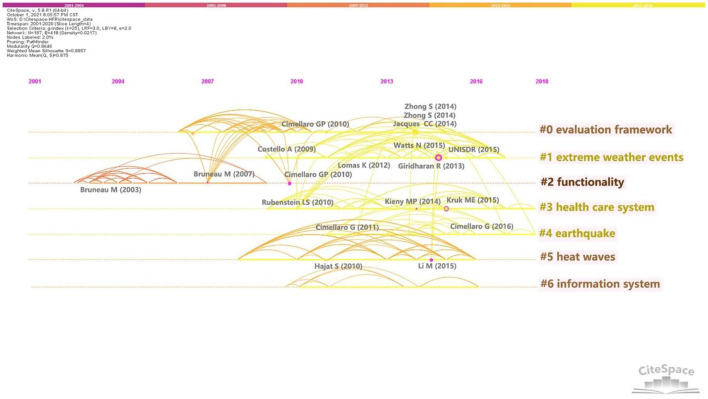
Timeline view of co-citation clusters.

Furthermore, the timeline view (Figure 7) reflects the evolution of HFR knowledge, in which the co-citation outbreak of each cluster was shown explicitly. The nodes of high centrality are marked by purple circles. The larger the node, the more times the document is cited (38). The more highly cited articles in a cluster, the more important the cluster is. In recent years, no HFR literature with high citation frequency has been found; thus, citation frequency needs time to accumulate (38, 39). Given the impact on the evolution of HFR knowledge, the literature with high centrality and high citations should be paid attention to. Linking different clusters can also be a potential turning point (34). Furthermore, in different clusters, an article may be highly cited literature and highly citing literature. *Seismic resilience of a hospital system*, published by Cimellaro et al. (40), is an example. It is highly cited literature at #0 and the highly citing literature at #6. Such articles can also be a potential turning point.

On the basis of the main research content, seven clusters are classified into four knowledge domains (Numbered as KD1-KD4) as follows.

(1) KD1 “climate change” = cluster #1 “extreme weather events” + cluster #5 “heat waves”

The knowledge domain KD1 focuses on the impact of extreme weather (heat waves, low temperatures, floods, and hurricanes) on healthcare facilities. Measures to reduce the negative effect of climate change are also discussed. Of all the extreme weather, scholars are mainly concerned with extreme heat due to serious climate change problems. This phenomenon can also be seen from the cited and citing references in this domain.

The most cited article in this domain was published by Lomas et al. (41), who proposed the renovation plans for the British hospital wards to cope with the elevated temperatures and achieve energy savings. In other highly cited articles, Short et al. and Lomas and Giridharan, worked on adapting hospital buildings for climate change with the application of data monitoring and building modeling technologies (42, 43). Hajat et al. and Nitschke et al. discussed the effects of heat waves on human health and the increased demand for healthcare facilities (44, 45). Morbidity, demand for ambulance services, demand for emergency medical services, hospitalization rates, and other indicators can be used to measure the impact on human health (14, 44, 45). Furthermore, indicators such as demand for ambulance services, hospitalization rates, and demand for emergency medical services can also be used to reflect the impact on health facilities (44, 45). The most citing article was published by Chand and Loosemore in 2016 (46), which discussed the vulnerability of healthcare facilities in geographical environment, built environment and organizational management under extreme weather. Six improvements were proposed to strengthen disaster management and improve HFR. Additionally, Curtis et al. (14) discussed the impact of climate change on the health and social care system given the healthcare services' provision and demand. Possible resilience enhancement measures, such as renovating hospitals to improve thermal comfort and increasing capacity for population risk identification and health awareness, are also discussed (14). Codjoe et al. analyzed the vulnerability of healthcare facilities and health services to flood and heat wave, and proposed measures to improve HFR in low-income areas (47). Extreme weather can cut off power supplies to healthcare facilities, which will disrupt the continuity of healthcare provision (48). Therefore, power outage problems have attracted the attention of researchers (49–51). A series of response measures were put forward, including preparing emergency generators (48, 50), better detection of power supply failures (49), the rise of distributed energy (52), and microgrids (53), etc.

From the timeline view (Figure 7), since 2008, the knowledge domain KD1 has begun to appear in highly influential articles. In 2009 and 2010, Costello et al. (54) and Hajat et al. (44) discussed the impact of climate change on health and the impact of surging demand on health care facilities. In 2012 and 2013, Lomas et al. (41), and Giridharan et al. (55) discussed the coping measures of hospital buildings and hospital spaces to elevated temperatures. In 2015, Li et al. discussed the impact of heat waves on morbidity and pointed out the need to establish data collection and monitoring systems to guide actions against climate change (56). In the same year, Watts et al. proposed a range of policy responses to climate change (57). *Sendai Framework for Disaster Risk Reduction 2015-2030* was proposed in 2015, in which public health and climate change are important parts (58). It can be seen from the recent citing literature that people in recent years are paying more attention to the energy issues and sustainability issues induced by climate change (52). With the frequent threat of global climate change, the term “resilience” is increasingly discussed in healthcare facilities' operation areas around climate change adaptation and disaster risk reduction (59, 60). A series of resilience measures to climate change adaptation are studied in recent years, including enhanced energy supply management, establishing early warning systems to collect climate and morbidity information, ensuring environmental sustainability of healthcare facilities, etc. (14, 57, 61).

(2) KD2 “strengthening resilience in response to war and epidemic” = cluster #3 “health care system”

This knowledge domain focuses on the impact of wars and epidemics on healthcare systems and measures to improve resilience. In particular, of all the wars and epidemics, the Syrian war and the Ebola virus outbreak are the two major disasters discussed in HFR research.

Epidemic and war lead to a shortage of resources and a rise in mortality. Furthermore, these two disturbances can often be combined to disrupt the supply of healthcare services. During the war, the use of violence to destroy healthcare facilities is increasingly common (62, 63). The destruction of roads, the breakdown of communications, and the threat of death from the war made it difficult for healthcare facilities to obtain adequate resources timely and prevented patients from being able or afraid to go to hospitals for treatment (64–66). The scarcity of health care services and the increasing number of patients further promote the outbreak of epidemics and ultimately lead to the collapse of healthcare systems (65, 66). In this case, many scholars have studied how to improve HFR to ensure the accessibility of healthcare services during wars and epidemics. The most cited article in this domain is published by Kruk et al. in 2015, who pointed out that a resilient healthcare system should identify threats as quickly as possible, be multi-layered, and have broad coverage (67). Furthermore, a resilient healthcare system should have strong links and adequate communication with external organizations or countries (67, 68). During outbreaks of war and infectious diseases, the concept of resilient health systems has been studied in greater depth (69). People pay more attention to the positive role that individual facilities within the system can play (69). In highly cited articles, Gilson et al. (70) and Kieny et al. (71) also believe that giving full play to the role of leaders in the healthcare system, and strengthening the information exchange within and outside the healthcare system will be powerful measures. In the most citing literature, Jamal et al. (66) pointed out that providing physical and psychological support to employees, ensuring organizational flexibility, and establishing good collaboration and communication mechanisms are key points. In the highly citing literature published by Douedari et al. (72), strategic vision, participation, transparency, responsiveness, equity, effectiveness accountability were considered as critical elements for healthcare system governance. In addition, Fouad et al. (62) and Raven et al. (73) stated it was necessary to provide a safe working environment and appropriate incentives for front-line health workers (62, 73). These measures will help them relieve the psychological pressure, and subsequently the continuity of healthcare provision can be ensured to some extent (71). In addition, many cited articles (67, 68) and citing articles (62, 74) emphasized the necessity of establishing an information monitoring system, which is important for the prevention and control of epidemic outbreaks.

The timeline view (Figure 7) shows KD2 active since 2009. A highly cited paper in this knowledge domain was published by Rubenstein and Bittle (75) in 2010 which highlighted the importance of protecting healthcare facilities and medical staff in conflict and discussed the possible effective strategies. In 2014 and 2015, nodes with high centrality appeared. These two articles were published by Kieny et al. in 2014 (71) and Kruk et al. in 2015 (67), respectively. They all discussed the impact of the Ebola virus outbreak on healthcare systems and measures to improve resilience of systems. This may be related to the spread of Ebola virus from 2014 to 2015. The content of discussion in this knowledge domain does not evolve and change obviously.

(3) KD3 “resilience assessment” (ND3) = cluster #0 “evaluation framework” + #2 “functionality” + cluster #4 “earthquake”

In this domain, researchers applied various approaches to assess HFR and proposed several measures to improve resilience in the face of disasters (6). “Evaluation framework” consist of a number of resilience measures or indicators for resilience measurement (15, 76). “Functionality” is the most commonly used metrics for describing HFR (77). Particularly, the earthquake is the popular disaster discussed in this knowledge domain.

In the most cited literature, Jacques et al. (78) in 2014 used fault tree analysis to identify factors that affect critical hospital services, namely, non-structural component failures and external utility supply disruptions. In addition to the above two factors, Mitrani-Reiser et al. (79) and Kirsch et al. (10) also considered the damage to structural components, equipment and workers. Achour et al. (80) focused on the influence of public utilities, in which the impact of utility disruptions on healthcare facility was quantified. Some research utilized the modeling-based approach to assess HFR. For example, Cimellaro et al. (3) in 2011 and Cimellaro et al. (8) in 2016 proposed meta-models and discrete event simulation (DES) to assess resilience in the emergency department. In these studies, “patient waiting time” was used as a final measure of resilience (3, 8). Furthermore, the most citing article was published by Zhong et al. (15) in 2014, who developed a framework with eight areas to measure the resilience of hospitals to disasters. On this basis, factor analysis was conducted to extract four key capabilities (including emergency medical response capability, disaster management mechanisms, hospital infrastructural safety, and disaster resources) to measure HFR (15). In the same year, another framework was proposed by Zhong et al. (21) using literature review method. The key assessment areas are classified as robustness, redundancy, resourcefulness and rapidity. The functionality or performance of healthcare facilities is a commonly used resilience measure in many HFR assessment methods (77). In the highly cited articles, Cimellaro et al. (81) and Cimelaro et al. (40) quantified HFR by considering the loss and recovery phases of a healthcare facility's functionality. Furthermore, Khanmohammadi et al. (6) simulated the dynamic recovery process of hospital function after earthquakes. This model can help decision-makers assess the resilience of post-earthquake hospitals and determine the optimal use of available resources (to help hospitals recover their functions) (6). The citing article, Cimellaro et al. in 2019, used “patient waiting time” to measure the performance of a healthcare facility network in emergencies and proposed two resilience improvement strategies, namely, reallocating existing resources and building new emergency departments (82).

From the timeline view (Figure 7), the citation phenomenon appears among #0 “evaluation framework,” #2 “functionality,” #4 “earthquake,” which is sufficient to illustrate the intimate relationship between these three clusters. Among the three clusters, #2 “functionality” appeared the earliest among the top seven clusters, in which the citation outbreak appeared since 2003. The first widely cited article in this domain was published by Bruneau et al. (83) in 2003. In this study, “4R,” namely robustness, redundancy, resourcefulness, and rapidity, is used to summarize the concept of resilience for the first time (83). Since then, scholars have proposed many ways to evaluate HFR. Bruneau and Reinhorn (84) in 2007, Cimellaro et al. (81), and Cimellaro et al. (40) used probability functions to quantify the loss and recovery of performance/function in healthcare facilities. It is also common to identify key indicators and develop a framework model for assessment by combing the key capabilities of HFR (15, 21). Furthermore, scholars tend to evaluate HFR by modeling the interactions and behavioral changes of sub-systems within a facility, such as Cimellaro et al. (3) in 2011 and Cimellaro et al. (8) in 2016. With the deepening of the research, the research content gradually expanded from individual healthcare facilities to healthcare networks or systems. In 2010, Cimellaro et al. (40) assumed that the performance of healthcare networks was simply equal to the aggregation of individual facilities' performance. Based on an empirical study, Jacques et al. (78) in 2014 further discussed the positive effect of service sharing among hospitals on healthcare network performance. A recent citing article (85), which Hassan and Mahmoud published in 2020, established a framework model for assessing the resilience of post-earthquake healthcare systems. This model considered the quality and quantity of services provided by the hospitals, the demand and arrival rate of patients on the hospitals, and the interaction between hospitals and other infrastructures (85). HFR assessment studies are increasingly complex and profound.

(4) KD4 “applications of information system” (ND4) = cluster #6 “information system”

This knowledge domain mainly discusses the application of technologies in the healthcare industry. With the help of these technologies, healthcare facilities can process large amounts of information quickly, and provide more efficient, higher-quality services to more patients in less time.

A common application area with technologies is optimizing the layout of healthcare facilities. For one thing, in resource-constrained settings, well-located facilities can greatly improve access to health services, ensuring accessibility at the community or city level while making full use of resources. The most cited article (86) by Ferguson et al. in 2016 used Geographic Information System (GIS) to identify critical and efficient paths for patients accessing health services. Healthcare facilities are prioritized at the sites with large population congregations and intersection of these paths (86, 87). For another, with the help of GIS, the location of healthcare facilities can avoid low-lying areas, flood and other disasters or reduce the probability of such events (47). Another application area discussed frequently is decision support for resource allocation. It is reflected in prioritizing the provision of resources to core functional components to ensure service provision, and facilitating the reasonable matching of resources and demands to speed up the recovery of facility performance. In an article published in 2010 by Paturas et al. (88), a Hospital Emergency Support Functions (HESF) model based on the personnel database was developed urgently to review all functions and staff in the hospital. The most suitable personnel will be assigned to key positions based on the importance of the function and the capacities of the people. Additionally, medical diagnosis and treatment is popular in recent years. The COVID-19 outbreak has boosted popularity of telemedicine technologies. As the citing article (18) by Haldane et al. in 2020 and the citing article (89) by Bhaskar et al. in 2020 pointed out, telemedicine can ensure physical distance between people, reduce the route of transmission of the virus, and provide health services continuously.

From the timeline view (Figure 7), the knowledge domain KD4 began to rise around 2010, which is a relatively new research topic. The development of technologies also takes time to accumulate. These may be the reasons why this domain has no large citation outbreak and high centrality nodes. In recent cited literature, COVID-19 is an unshirkable research content (18, 90). They illustrate the impact of epidemics outbreaks on the use of technologies in healthcare facilities. With the advent of the data age, the everchanging science and technology will make this field of knowledge active for a long time.

## Discussion

**Figure 8:**
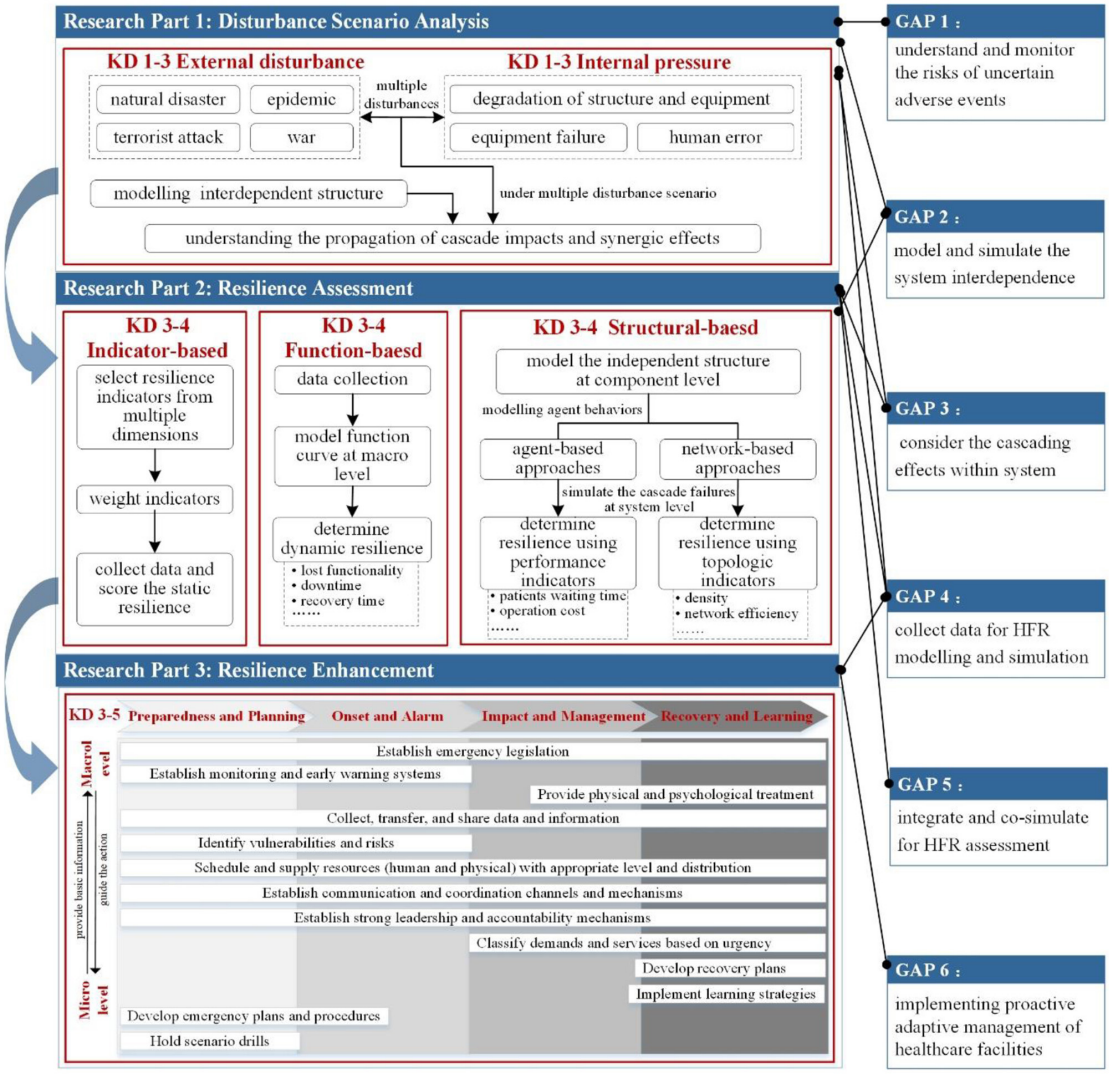
Knowledge map of HFR.

Despite the considerable amount of HFR studies, no earlier effort has been given to existing aggregate findings quantitatively and comprehensively to our best knowledge. Thus, a comprehensive knowledge map for HFR is put forward (Figure 8) based on the aforementioned scientometric review and deep content analysis. The following part discussed the critical research parts, current research gaps, and future work.

### Critical Research Parts of HFR

As shown in Figure 8, this framework depicts three critical research parts of HFR: disturbance scenario analysis, resilience assessment, and resilience enhancement. Their states are described as follows.

#### Disturbance Scenario Analysis

When addressing the HFR issues, the researchers must primarily clarify what the healthcare facilities are resilient to (91). Specifically, which resilience strategies should be followed depends on the specific country context and the type of disturbances (12). Different economic-level countries focused on different disasters due to their specific regions, various healthcare level and infrastructure conditions, and diverse political conditions (92). Though content analysis, we stratified the data (collected articles) with regard to the involved countries with different economic level and their concerned disasters (Table 1) (93). Of the total 55 involved countries, there were 17 high-income countries (30.91%), 11 upper-middle income countries (20%), 17 lower-middle income countries (30.91%), and 10 low-income countries (18.18%), respectively. It appears that the healthcare sector of these countries is largely focused on acute shocks. High-income and middle-income (upper-middle and lower-middle) countries are more concerned about climate change and natural disasters, such as earthquakes, hurricanes, floods, etc., while low-income countries mostly discussed about Ebola and conflicts. Recently, with the emergence and rapid spread of COVID-19, more studies are trying to discuss the national response experience of the high-income countries and upper-middle income countries, and aiming to draw lessons from healthcare system that have proved more successful (12). Clearly, climate change issues, natural disasters and epidemics will severely test the healthcare facilities in all countries around the world in future. Understanding the pathways of various disturbances not only helps decision-makers prepare for problems but also manage better when they happen.

**Table 1:** Data stratification based on countries' economic level and concerned disasters.

**Disasters**	**Low-income countries**	**Lower-middle income countries**	**Upper-middle income countries**	**High-income countries**
Heat wave	1	2	1	6
Low temperature				3
Flood	2	5	3	8
Hurricane		3	2	9
Earthquake		5	6	22
Tsunami		1		2
General climate change issues/natural disasters	3	16	7	25
COVID-19	1	1	5	10
Ebola	18	1	6	3
General epidemics	5	5	6	4
Refugee	2	2		
Conflict	10	1	3	
Terrorist attack	2		2	1
Economic crisis		1		2
General disasters	1	2	9	19

Disturbance scenario analysis characterizes the type, severity, occurrence time, and potential risks. For healthcare facilities, the disturbances are classified into external disasters (e.g., earthquakes, large floods, epidemic, and war) and internal pressures (e.g., equipment failure and human error) (47, 62, 94). The external disasters will cause the healthcare facilities' internal structural failure directly or interrupt the supply of external lifeline services (e.g., electric, water, communication, and gas) for healthcare facilities. Evidence shows that disasters are not always isolated events and usually occur in complex combinations (95). For instance, in 2008, the Wenchuan Earthquake in China was followed by floods and landslides (96, 97). Following such complex disturbances, healthcare facilities face significant operation challenges regarding the continuity of healthcare service (80). Considering the different disturbance scenarios, Zhao et al. and Ouyang et al. presented two basic scenario modes: single-disruption scenario and multiple-disruptions scenario (98, 99). The latter mode shows the impact of the initial event and its ensuring events on the performance of the infrastructure system. Some studies have focused on the single disturbance event in terms of healthcare facility resilience. For example, Bruneau and Reinhorn (84) in 2007 discussed seismic resilience in acute care facilities. Few studies began to consider the multiple disruption scenarios in the healthcare facility context. For instance, Qirbi and Ismail (100) in 2017 discussed the impact of wars and epidemics on healthcare facilities in Yemen.

Furthermore, the disturbance scenario analysis should understand how the disturbances influence the system (101). Such analysis should involve analyzing the system structure and clarifying the initial and subsequent failure caused by disturbances. The healthcare facility can maintain its normal function owing to external lifeline services (e.g., the municipal water supply, electric power supply, and gas supply) and the internal equipment systems (e.g., electric power system, HVAC system, water supply system, and gas system). In addition to these physical aspects, the healthcare facility operations also require organizational and social efforts. Healthcare organizations, including medical care departments, facility management department, asset management, department, and administrative department, operate numerous facilities serving core business relating to medical care, research, laboratories, and education (102). Social units refer to the government sector, fire service, police department, and social media that provide rescue services and resources for the healthcare facilities to deal with emergency events (83). All these dimensions are not isolated but highly interconnected and mutually interdependent (101, 103, 104). For example, water supply systems require an electric power supply to maintain their normal operations, whereas the electric power system needs water resource for power delivery. Thus, modeling and analyzing the interdependent system structure of healthcare facilities is a critical step for disturbance scenario analysis. Given the interdependencies, the cascading failures occur across the systems when the disaster event happens (80, 104, 105). Specifically, one sub-system's failure or function loss may lead to knock-on consequences in others and eventual collapse of the entire system. These cascading failures will be more serious under multiple disturbances. Thus, a full understanding of the propagation of cascade impact and synergic effect in the healthcare facility systems is necessary.

#### Resilience Assessment

“Measurement” is needed to identify what needs to be improved (106). Resilience assessment plays a decisive role in determining the strategies for resilient healthcare facilities development (107–109). Given this significant role, most previous HFR studies have focused on quantifying or assessing resilience. This section categorizes and reviews the existing resilience assessment methods for healthcare facilities. They are broadly grouped into three types: indicator-based, function-based, and structure-based approaches.

In terms of the indicator-based approaches, most studies proposed a generic framework for HFR assessment, which integrates several dimensions with a set of resilience measures (110). The critical steps for these approaches commonly include selecting and categorizing resilience indicators, weighing each indicator's importance, scoring the indicators, and calculating the final resilience results. For example, Bruneau et al. proposed a general framework to evaluate the seismic resilience of any physical and organizational systems (83). This framework quantifies resilience with four properties: robustness, redundancy, resourcefulness, and rapidity (83). Various studies have been implemented to quantify resilience from different perspectives to support decision-making. For example, Cimellaro et al. (111) in 2018 used a questionnaire and factor analysis to identify three resilience factors, including cooperation and training management, resources and equipment capability, and structural/organizational operating procedures. A linear combination of the three factors was eventually used to represent HFR. In sum, the indicator-based approaches capture the characteristics of multiple dimensions for HFR, and implementing these approaches is easy. However, most selected resilience indicators in the indicator-based approaches are static and cannot measure HFR in a dynamic way. The resilience capacities of healthcare facilities are process-based, which are reflected by the healthcare facilities' dynamic responses to disruptions.

The function-based approaches provide a quantitative and direct means to evaluate HFR by using the performance curve describing the functionality of healthcare facilities. Collected from the time-dependent performance curve, several macro-level indicators were used to determine dynamic resilience, such as lost functionality, downtime, and recovery time. For example, Cimellaro et al. (3) in 2011 defined the disaster resilience of a hospital as the product of technical and organizational resilience. Patient waiting time was used to measure organizational resilience. The number of untreated patients vs. the total number of patients requiring treatment, that is, healthy population loss, was used to measure technology resilience. One challenge with these approaches is that healthcare facilities' critical functionality/performance in scenario time is difficult to define (103). Common sense and previous studies indicated that the functionality of healthcare facilities can be defined in terms of quality service. For instance, Cimellaro et al. (40) in 2010 expressed seismic resilience by the patients' waiting time for treatment as an index of service quality. More attributes, such as losses, recovery cost, and time, were further selected to measure the resilience to recover from losses generated by the earthquake (40). In the case of COVID-19, the critical functionalities of healthcare facilities are to hospitalize all infected persons and continue to provide normal care for non-COVID-19 patients. Accordingly, the number of intensive care beds available and the available personal protection equipment/resources are specifically used to quantify resilience of the healthcare facilities (103). Thus, the definition of system functionality and corresponding resilience indicators will be changed according to the facing disasters. Therefore, the function-based approach can dynamically determine the resilience by comparing the macro-level functionality/performance of healthcare facilities before and after disruptions in time scenarios. However, these approaches did not take the healthcare facilities' micro-level system structures and behaviors into consideration. Consequently, it is difficult to dig out the vulnerable and critical components of the healthcare facilities and develop the targeted resilience enhancement strategies.

The structure-based approaches considered the system structures and their impact on resilience in healthcare facilities (101). The healthcare facilities' response to disturbances can be represented by modeling the system structures at the component level and then simulating the cascade failures at the system level. Several indicators from the simulation model can be used to measure the resilience level, such as many waiting patients and admission rates (6, 85). Depending on whether the behaviors of decision-makers are modeled, the present study characterizes structure-based approaches into two types: agent-based approaches and network-based approaches. Agent-based models are often used to simulate the actions and interactions of agents (112, 113). In 2001, Taboada et al. (113) modeled a hospital emergency department using a proxy-based approach, in which the changes in patient waiting time were shown under different patient arrival rates and different types and numbers of ED staff. As for the network-based approach, nodes are used to represent critical components of the system, and the relationships between nodes are represented by links (112). According to whether modeling the practical flow within systems, network-based methods can be divided into topology-based methods and flow-based methods (41). For example, Akcali et al. (114) in 2006 established a network flow model to optimize the bed capacity in a hospital under the premise of minimum cost. Arboleda et al. (101) presented a network flows model to assess the vulnerability of a healthcare facility during disaster events, considering the flow of patients within the facility, the interactions between the facility's different service areas, and the external lifeline services. In sum, the structure-based approaches capture the system's topological features and even simulate the participants' behaviors on these topological structures using agent-based technologies. The resilience mechanism can be explored by simulating the interactions between system structures, participants' behaviors, and disasters. Thus, effective strategies on resilience enhancement can be simulated in these structure-based models, and the optimal resilience enhancement strategies would then be selected. However, concerning the accessibility of data for modeling and simulation, the structure-based approaches, particularly the agent-based methods and the flow-based methods, are difficult to access the required data. This limitation is serious in the healthcare sectors. The most complex data sets, such as the human behavior variables and the facilities' operational data, are difficult to obtain because of privacy and security issues (112).

#### Resilience Enhancement

The resilience enhancement of healthcare facilities is a sophisticated and systematic process, for which the type and severity of disturbances, the stage of the disturbance, the healthcare facilities conditions as well as the specific country context should be fully considered (12, 110). Generally, resilience enhancement strategies are provided later after resilience assessments. For this, we directly distilled the resilience strategies from existing research results. The following section will depict these resilience strategies at different stages of the response cycle, in which macro level (healthcare systems) and micro level (single healthcare facility) were both involved. Moreover, some resilience strategies could be conducted across stages. Here, we map specific strategies to particular stages of disaster response in order to highlight their critical relevance in these stages.

##### Stage 1: Preparedness and Response Planning

Resilience to acute disturbances is enhanced by adequate preparedness (46, 110). The preparedness stage relates to reducing the vulnerability of the healthcare facilities to various disturbances (46). At this stage, general preparation for any disturbances includes response planning for possible threats, resourcing those plans and holding scenario drills as planned (46). It's important to note that healthcare facilities vary widely in the degree to which they prepared for the range of possible disturbances (110). The degree of preparedness will be determined by the frequency and severity of the possible risks. Threats with high probability or high impacts should be given priority in preparedness and response planning. However, building too much preparedness for a specific disaster might increase the healthcare facilities' vulnerability to other unanticipated threats due to limited workforce and resource (12). Thus, anticipating possible disturbances and ensuring sufficient resource with adequate distribution are critical element of preparedness.

Learning from previous studies, the micro-level preparedness in single healthcare facility is summarized as the following areas: assessing the healthcare facility's structural and non-structural vulnerabilities, mapping the intensity and probability of possible threats, setting emergency plan and protocols, preparing emergency teams and assigning of responsibilities, training for emergency response procedures, and ensuring human and physical resources sufficient with appropriate level and distribution, etc. (15, 21, 46, 110, 115). The macro-level preparedness mainly relates to how well a country/region prepares for future disturbances affecting its healthcare system (12). Examples of preparedness and response planning have been specified as critical for resilience, including establishing strong leadership and accountability of government agencies for emergency response, developing coordination channels and data-sharing mechanisms across government and key stakeholders, and ensuring sufficient healthcare system resources (healthcare related resource and critical infrastructure support) and mobilizing all available resources across regions for deployment in future threats, etc. (66, 72, 74, 92). Clearly, healthcare system preparedness provides communication channels and governance mechanism for information sharing and resource support across healthcare sectors, different levels of government and the other social sectors (i.e., media, community committees and infrastructure service) in the case of emergency crisis (12). It is also noted that preparedness in single healthcare facility provides a backbone for developing and implementing national/regional preparedness and response plans (116). Specifically, single healthcare facility provides front-line data including vulnerability risks, resource requirements and service capacity, etc. (117). These data underpin effective decision-makings for developing national/regional preparedness and response plans, which includes anticipate external threats and identify internal vulnerabilities of healthcare system, clarify existing gaps between service supply and demands at national/regional level, and determine the appropriate level and distribution of resources across healthcare system (67). In addition, single healthcare facility is at the core position for supporting the implementation of national/regional plans and ensuring the continuous service delivery in response to threats (66).

##### Stage 2: Disturbance Onset and Alarm

The focus of this phase is on early identification of the onset and type of the disturbance (12). Clearly, the earlier that the disturbance is noticed, the faster and more effective the response actions can be. An effective surveillance and early warning system would be a powerful tool at both macro level and micro level (74, 90). Take the epidemic threats as examples, surveillance applications include detecting the abnormal increase of the case, monitoring and describing the magnitude and patterns of infectious disease, predicting epidemic trends, and discovering the emerging infectious disease (118). Then, early warning releases the signals to relevant institutions and personal for further control actions. Critically, effective surveillance and early warning system builds primarily on active data collection and information-sharing mechanisms (71, 74). Particularly, as the basic data resources, the hospitals, clinics and community care centers should take the responsibility of collecting reliable data and reporting to relevant sectors in time (67). In view of healthcare systems, it is suggested to expand the data scope and build trans-city or even transnational information monitoring systems to achieve large-scale communication and information sharing (74).

##### Stage 3: Disturbance Impact and Management

This stage places greater emphasis on the ability to absorb the impact of initial damage, minimize adverse consequences and ensure the continuous service delivery (98). Generally, threats will disrupt the balance between supply and demand of healthcare service. And the shortage of health professionals and resources is serious. Experience from previous crises, increasing service capacity and adopting alternative and flexible approaches to ensure continuous healthcare delivery are strong need for strengthening resilience when healthcare facilities were shocked by threats (12). A series of specific response strategies for single healthcare facility were explored from previous studies, including triaging patients and treating them according to their urgency level, giving priority to maintaining healthcare service delivery, shifting operation activities to lower-cost settings, activating the backup resources (i.e., health professionals, medical resource, lifeline service) for increasing service capacity or preventing service disruptions (6, 8, 80, 110). The response actions of healthcare system will promote absorption and adaption abilities at strategic level. Effective information systems are critical for decision-making across healthcare system (74). For example, based on the flow of data and information across healthcare system sectors, decision-makers can precisely match the demands of healthcare and resources with available supply in view of fast response and transfer cost (78, 79, 85). It is noted that these resource sharing and coordination activities should be underpinned by emergency legislation at national/regional level (57). Additionally, transparent communication to the public, and creation of public trust and support are also crucial in response to emergencies (12).

##### Stage 4: Recovery and Learning

In this stage, the focus is on taking a series of adjustments to better recover from the impact of the disturbance and return to some kind of now normality (12). The post-shock context has to deal with several legacy issues, such as lost estimation, recovery decision-makings, and rebalance of the demand and supply, etc. (6, 119). For the micro-level single healthcare facility, several studies proposed specific strategies for recovery decision-makings. For instance, a decision support platform was developed for determining the priority of the damaged components and allocating recovery resources in consideration of both recovery time and cost (7, 57, 84). Ouyang et al. proposed that developing efficient communication channels and coordination mechanisms for rapid recovery response is necessary (93). In addition, other strategic legacy issues have to be done by macro-level healthcare systems. For example, scheduling recovery resources across regions or sectors and the long-term of physical and psychological treatment after shocks are great challenges (72, 74). Critically, all these decision-makings for recognizing legacy aspects and figuring out operable recovery strategies rely heavily on data collection (e.g., loss data, repair time, and required amount for recovery resources) and data analysis methods. Furthermore, learning from success and failures and adaptation to future is vital for building resilience (92). Several learning strategies could be noted for healthcare facilities, such as developing organizational learning culture and developing mechanisms to conduct feedback analysis and experience summary (46). In essence, most resilience strategies across the disaster response stages were summarized through learning from past experience.

### Knowledge Gaps and Future Research

Reviewing the articles relating to HFR in 2000–2020, this study identifies the areas that are still requiring further studies. As Figure 8 shows, these areas are summarized into the knowledge gaps of HFR and set the directions for future research.

The first knowledge gap is related to understanding and monitoring the risks for uncertain adverse events. The resilience concept emphasizes the resistant capacity of the system to understand and prevent any possible hazards (including emerging risks) (98). In terms of the functionality of healthcare facilities, monitoring the risks of public health and the loopholes of the healthcare systems are essential for the healthcare facilities' disaster prevention (67). The traditional monitoring systems usually collected public health data from clinical cases, which are labor-intensive and time-consuming (120–123). This method is inefficient for dealing with the emerging risks, particularly the epidemics with a fast spread and long incubation period (103). Taking COVID-19 for example, in some countries without powerful healthcare support and warning mechanisms, an average delay of 29 days was observed from the onset of symptoms to the detection of the epidemic outbreak (124). This delay finally resulted in the widespread COVID-19 and huge pressure on healthcare systems. Thus, studying the hazard monitoring system for healthcare facilities is urgent. Specifically, monitoring the data of climate change (e.g., the rising temperature and possible floods) and exploring their impact on public health can be noted for the early identification and monitoring of public health events (56). Furthermore, given that the clues of risks are probably hiding in the public, how to use the data from social media for monitor the public's reactions is also worth further research, in which collection methods and data cleaning technologies are critical points.

The second knowledge gap is the modeling and simulation of system interdependency. A range of recent HFR studies discussed the impact of disturbances on healthcare facilities (14, 43, 80), but few studies can use the “structure-based” method to quantify the resilience level of healthcare facilities (112). The main reason for this limitation is that existing studies do not fully support modeling and simulation of system interdependency and cascading effects for healthcare facilities (112). In terms of system interdependence, the operational healthcare facility and its emergency management heavily depend on external lifeline services, internal equipment systems, healthcare organizations, and social units (80, 125). All these dimensions are highly interconnected and mutually interdependent. In disasters, the performance deterioration of healthcare facilities can be easily amplified because of interdependencies. Thus, for HFR research, future research would characterize the structure of complex healthcare facility systems and identify approaches to simulate this interdependent system.

The third knowledge gap is related to considering the cascading effects of healthcare facilities. How disturbances cascade through the interdependent systems has to be assessed to estimate the performance deterioration and mitigate the consequences of failures (125). Some studies proposed the methods of revealing cascade failures for interdependent critical infrastructures. For example, Utne et al. developed a cascade diagram to represent the cascading failures across the interdependent critical infrastructures under the accident scenario (126). Lam and Tai integrated network and fuzzy set theory to reveal the cascade effect from a disruption (127). Ouyang reviewed the approaches of modeling interdependent critical infrastructure systems, including empirical approaches, agent-based approaches, and network-based approaches (112). Those studies focused on the general essential infrastructure systems, such as the power grid, telecommunications, transportation, and water supply systems. However, in-depth research on modeling healthcare facility systems is still lacking. Furthermore, these proposed modeling methods for general infrastructure systems can also provide directions for HFR studies in the future.

The fourth knowledge gap is concerning the data collection for HFR modeling and simulation. All proposed resilience assessment approaches require a lot of relevant data, such as the healthcare facilities' system structures, organizational structures, operational data, emergency procedures, performance data, experts' experience, and historical events' data (112, 128). To collect these data is generally difficult because of a series of reasons. For example, much historical data on previous disturbances in healthcare facilities are incomplete and imprecise because of the backward information recording and preservation methods or awareness (129, 130). This limitation directly blocked the use of function-based approaches for empirical resilience assessment and data basis to validate other modeling and simulation methods (112). Furthermore, the healthcare facilities' operational data and their performance metrics are usually confidential because of safety issues or commercial secrets. Despite scholars and practitioners appealed to develop the data collection mechanism for events data in healthcare facilities, data collection or accidents reports for HFR studies have no standard. Thus, developing a unified database for monitoring and collecting the events data in healthcare facilities is essential. The following data standards and data analysis methods can be further researched for HFR studies.

The fifth knowledge gap is related to integration and co-simulation for HFR assessment. The previous review of resilience assessment approaches depicts that each approach has advantages and disadvantages. Function-based approaches can dynamically and directly reflect resilience capacities by the changing macro-level functionality/performance of healthcare facilities (40, 81). However, they fail to identify the critical components within healthcare facility systems that can significantly impact resilience because they did not consider the micro-level system structures, interdependences, and response behaviors (6, 128). Accordingly, the structure-based approaches only used topological indicators to represent resilience in a static way, which could not fully reveal resilience's “process-based” characteristics in a time scenario. To develop the strong points and avoid the weak points for HFR assessment studies, an open modeling framework to capture the macro-level performance indicators and the micro-level topological indicators is more desired for practical applications. This framework will involve integrating modeling and simulation methods for HFR, and specifically exploring the relationship between the micro-level topological indicators and the macro-level performance indicators in a mathematical way is necessary.

The sixth knowledge gap has to do with proactive adaptive management of healthcare facilities. Most existing studies give more efforts to existing function recovery, but little attention is devoted to recognizing the healthcare facilities as an adaptive system (92). After the disaster is completely gone, the system of healthcare facilities had been re-stabilized. In most cases, this stable state is different from the stable state before the disaster. This is due to the impact of disasters and measures implemented to ensure the functioning of healthcare facilities (12). Exploring differences between these two steady-states and their causes will be meaningful for discovering the pathways of strengthening future resilience in a system view (12). Additionally, tightly linking the recovery and learning experience to preparedness is crucial although often neglected once the function is recovered (12). Strengthening the resilience of healthcare facilities in an adaptive view is not only for improving the current system but is also better for response to future threats scenarios.

## Conclusion

Healthcare facilities are one of the most important and complicated critical facilities in any region and country. During disasters (e.g., earthquake, flood, and epidemic), their role is even critical for rapid and effective response to casualties, injuries, or infected patients. Thus, the resilience of healthcare facilities has gained much attention in recent studies. This scientometric review aims to detect the status quo and future trends of healthcare facility resilience research. After directional search and exclusion, 374 articles between 2000 and 2020, which were gathered from the WoS core collection database, MEDLINE database and Scopus database, were analyzed to explore and visualize the current status and future trends of healthcare facility resilience research.

In terms of the temporal distribution, research on HFR has experienced three stages: preliminary exploration period (2000–2007), slow development period (2008–2014), and rapid growth period (2015–2020). Most HFR studies originated from the USA, UK, Australia, Italy, and China concerning spatial distribution. Furthermore, the institutions that conduct HFR research are relatively scattered. Regarding the subject categories in co-occurrence analysis, engineering and public and environmental and occupational health were two major research subjects. Given the keywords, “resilience,” “hospital,” “disaster,” “health care,” and “healthcare facility” had the most frequency.

According to the literature co-citation networks, seven co-citation clusters were detected. This study classified them into four knowledge domains based on their contents: climate change impact, strengthening resilience in response to war and epidemic, HFR assessment, and information system applications. Furthermore, the timeline view of literature reflected the evolution of each domain.

Based on the aforementioned scientometric analysis, a knowledge map for HFR was put forward, in which the critical research contents, current knowledge gaps, and future research work were discussed. The critical research parts of HFR, including disturbance scenario analysis, resilience assessment, and resilience enhancement, were discussed in detail. Furthermore, knowledge gaps were identified in the areas of monitoring risk, modeling system interdependence, considering cascading effects, concerning data collection, HFR assessment barriers, and healthcare system resilience. Accordingly, the future research agenda were proposed: (1) studying the hazards monitoring system for public health based on climate change data and social media's reactions, (2) characterizing the structure of complex healthcare facility systems and exploring approaches to simulate this interdependent system, (3) modeling the cascading effects in healthcare facilities for estimating the performance deterioration and mitigating the consequences of failures, (4) developing a unified database for monitoring and collecting the standard events data for HFR research, (5) integrating and co-simulating the HFR assessment approaches for considering the micro-level topological indicators and the macro-level performance indicators, and (6) implementing proactive adaptive management of healthcare facilities.

This study provides an in-depth review of the status quo, knowledge gaps, and future research directions for researchers and practitioners in the HFR field. The proposed knowledge map of HFR is particularly useful for researchers to detect hot topics and find future research directions. Subsequently, the knowledge gaps will be filled, and the body of HFR knowledge will be extended.

## Author Contributions

LL conceptualized the research paper and contributed to manuscript writing. SL, JY, EW, and JS contributed to drafting the paper, data gathering, manuscript writing, data analysis and interpretation, and critical editing. All authors contributed to the article and approved the submitted version.

## Funding

This research was supported by the National Natural Science Foundation of China (Grant Number: NSFC-71901120 and - 72072031), and Humanities and Social Science Youth Foundation, Ministry of Education of the People's Republic of China (Grant Number: 19YJCZH080).

## Conflict of Interest

The authors declare that the research was conducted in the absence of any commercial or financial relationships that could be construed as a potential conflict of interest.

## Publisher's Note

All claims expressed in this article are solely those of the authors and do not necessarily represent those of their affiliated organizations, or those of the publisher, the editors and the reviewers. Any product that may be evaluated in this article, or claim that may be made by its manufacturer, is not guaranteed or endorsed by the publisher.
